# Associative-memory representations emerge as shared spatial patterns of theta activity spanning the primate temporal cortex

**DOI:** 10.1038/ncomms11827

**Published:** 2016-06-10

**Authors:** Kiyoshi Nakahara, Ken Adachi, Keisuke Kawasaki, Takeshi Matsuo, Hirohito Sawahata, Kei Majima, Masaki Takeda, Sayaka Sugiyama, Ryota Nakata, Atsuhiko Iijima, Hisashi Tanigawa, Takafumi Suzuki, Yukiyasu Kamitani, Isao Hasegawa

**Affiliations:** 1 Center for Transdisciplinary Research, Niigata University, Niigata-city, Niigata 951-8501, Japan; 2 Department of Bio-cybernetics, Faculty of Engineering, Niigata University, Niigata-city, Niigata 950-2181, Japan; 3 Department of Physiology, Niigata University School of Medicine, Niigata-city, Niigata 951-8501, Japan; 4 Department of Neurosurgery, NTT Medical Center Tokyo, Shinagawa-ku, Tokyo 141-8625, Japan; 5 Department of Electrical and Electronic Information Engineering, Toyohashi University of Technology, Toyohashi-city, Aichi 441-8580, Japan; 6 ATR Computational Neuroscience Laboratories, Keihanna Science City, Kyoto 619-0288, Japan; 7 Research Institute for Diseases of Old Age, Juntendo University School of Medicine, Bunkyo-ku, Tokyo 113-8421, Japan; 8 Lab of Neuronal Development, Graduate School of Medical and Dental Sciences, Niigata University, Niigata-city, Niigata 951-8501, Japan; 9 Center for Information and Neural Networks (CiNet), National Institute of Information and Communications Technology, and Osaka University, Suita-city, Osaka 565-0871, Japan; 10 Graduate School of Informatics, Kyoto University, Kyoto-city, Kyoto 606-8501, Japan

## Abstract

Highly localized neuronal spikes in primate temporal cortex can encode associative memory; however, whether memory formation involves area-wide reorganization of ensemble activity, which often accompanies rhythmicity, or just local microcircuit-level plasticity, remains elusive. Using high-density electrocorticography, we capture local-field potentials spanning the monkey temporal lobes, and show that the visual pair-association (PA) memory is encoded in spatial patterns of theta activity in areas TE, 36, and, partially, in the parahippocampal cortex, but not in the entorhinal cortex. The theta patterns elicited by learned paired associates are distinct between pairs, but similar within pairs. This pattern similarity, emerging through novel PA learning, allows a machine-learning decoder trained on theta patterns elicited by a particular visual item to correctly predict the identity of those elicited by its paired associate. Our results suggest that the formation and sharing of widespread cortical theta patterns via learning-induced reorganization are involved in the mechanisms of associative memory representation.

## Abstract (web summary)

Episodic or declarative memory is thought to be encoded in the ensemble firing of spatially distributed neurons. Here the authors use high-density electrical recordings to show that some areas in the primate temporal cortex develop patterns of theta activity that are similar for pairs of remembered objects.

## Graphical abstract

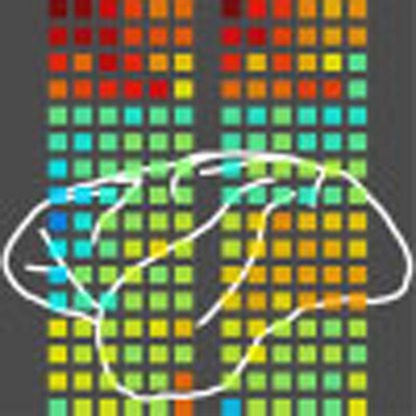

Declarative memory of episodes or semantic knowledge is not fragmented, but is linked by association to support our behaviour. Neuronal substrates of such associative memory have been explored in the temporal lobe of humans and non-human primates^1^,^2^,^3^,^4^. The encoding of associative memory depends on the interplay between the visual association area TE and the medial temporal lobe (MTL), including Brodmann areas 35 and 36 (A35 and A36), the entorhinal cortex (ENT), the parahippocampal cortex (PH) and the hippocampus (HC)^1^,^5^,^6^. Prevailing theories, including Hebb's cell assembly hypothesis, postulate that associated memory items are represented by mutually overlapping neural ensembles^7^,^8^. At the level of single neurons, recordings within macaque TE and A36 during visual pair-association (PA) memory tasks have revealed a class of neurons exhibiting discharges selective for learned associations^6^,^9^,^10^,^11^,^12^, which is especially prevalent in restricted regions in A36 (refs 10, 12). However, the entire picture of the cortical memory representation is still unclear. Importantly, whether memory formation accompanies area-wide reorganization of collective neural ensembles or just local microcircuit-level neuronal plasticity remains elusive, because of a lack of recording methods with high temporal resolution, wide spatial coverage and long-term stability. The collective activity is often observed to exhibit spectral modulation to facilitate neural computation, which is reflected in the temporal dynamics of local-field potentials (LFPs)^13^,^14^.

Here we hypothesize that associative memory is encoded by spatial patterns of specific LFP powers that are distributed across the temporal lobe. Therefore, we devise a novel high-density electrocorticographic (ECoG) electrode grid to acquire multi-site LFP recordings on the cortical surface, encompassing the inferior to the medial temporal lobes of monkeys performing a visual PA task^15^,^16^,^17^,^18^. We specifically test whether ECoG responses evoked by one item of a learned paired associate shares spatiotemporal patterns to those evoked by the other^19^. We show that transient theta (4–8 Hz) responses in TE, A36 and the PH, but not in the ENT, express similar spatial patterns specific to associated visual items. We further demonstrate that the spatial pattern similarity in theta activity emerges through novel PA learning. These results suggest that the formation and sharing of specific spatial patterns of theta activity are involved in the mechanisms of associative memory representation in the temporal cortex.

## Results

### Electrode grid implantation

**Figure 1:**
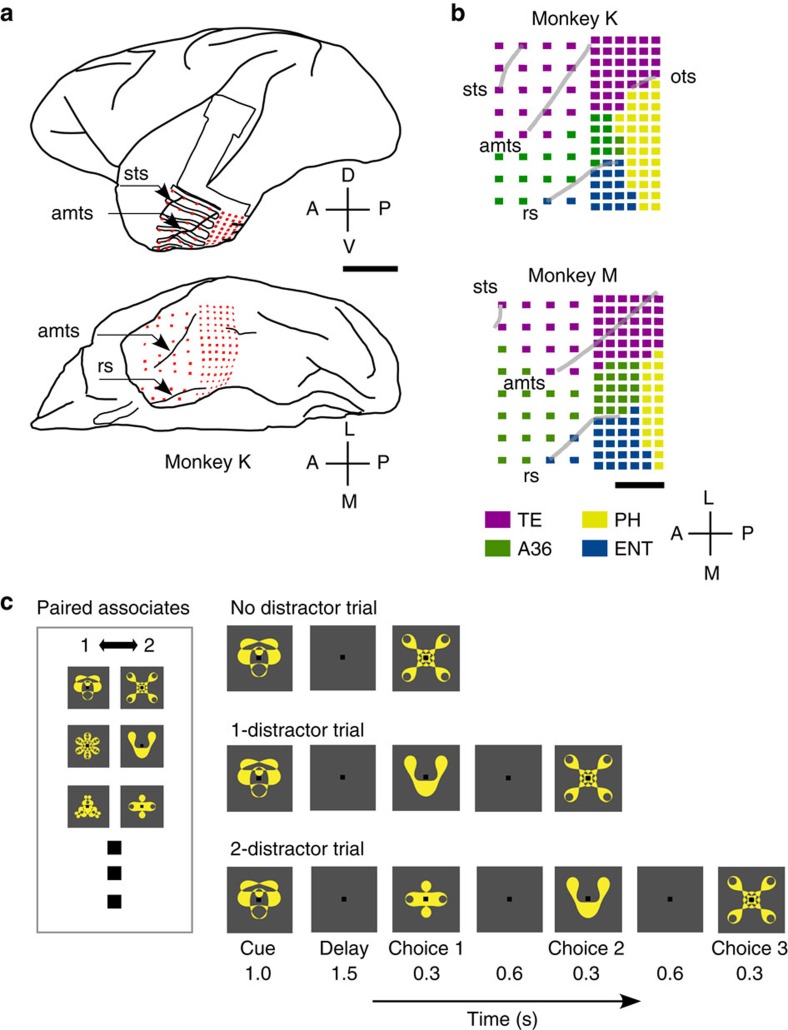
ECoG recording site and task procedure. (**a**) Lateral (top) and ventral (bottom; only the left hemisphere is shown) views of a monkey brain implanted with an ECoG electrode grid, reconstructed by post-mortem observations. Red dots indicate individual electrodes. amts: anterior middle temporal sulcus; ots: occipitotemporal sulcus; rs: rhinal sulcus; sts: superior temporal sulcus. A-P: anteroposterior; D-V: dorsoventral; M-L: mediolateral. Scale bar, 10 mm. (**b**) Recording site. Areas TE, 36 (A36), the PH and the ENT are labelled based on the histological analysis. Scale bar, 5 mm. (**c**) PA memory task. (left) Examples of visual stimulus–stimulus associations. (right) A visual stimulus was presented as a cue (1 s) followed by a 1.5 s delay, and up to three choice stimuli were sequentially presented. The monkeys were required to respond upon the presentation of the correct paired associate of the cue.

Two monkeys (*Macaca fuscata*) underwent subdural implantation of an ultrathin, 128-channel electrode grid over the anterior middle temporal sulcus (amts). The electrode grid configuration relative to the entire amts and the superior temporal sulcus (sts) was closely matched across subjects under intraoperative inspection (Fig. 1a,b and Supplementary Fig. 1). Moreover, we conducted post-mortem histological analyses to determine anatomical areal boundaries within the recording sites. We classified electrode channel locations based on the cortical cytoarchitecture underneath as revealed by Nissl staining and parvalbumin (PV) immunohistochemistry^20^,^21^. We confirmed that the electrode grid covered inferior TE, posterior A36, the lateral part of the ENT and the anterior part of the PH in the two monkeys (Fig. 1b and Supplementary Fig. 2).

### Memory retrieval task and cue-evoked ECoG potentials

**Figure 2:**
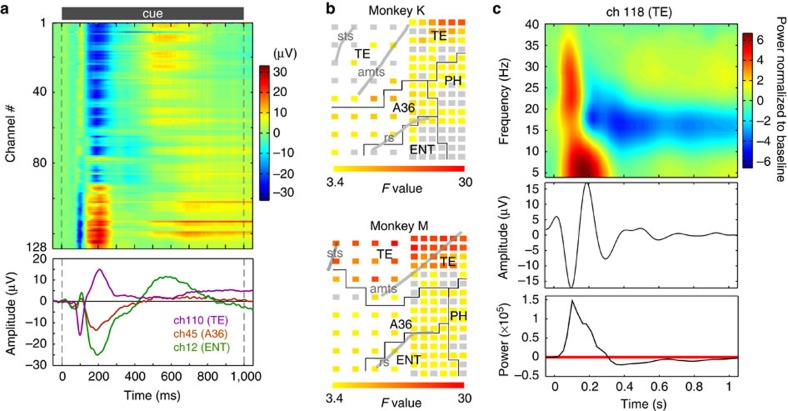
Cue-evoked ECoG potentials and stimulus selectivity of theta power. (**a**) Visual cue-evoked ECoG potentials. (top) Amplitudes of cue-evoked potential averaged across trials at each of the 128 channels in monkey M. Channel number assignments are shown in Supplementary Fig. 1. (bottom) Representative waveforms of cue-evoked potentials. (**b**) Channel-wise mappings of response selectivity of theta activity to the cue stimuli. *F*-values above significance level (*n*=2 monkeys, one-way ANOVA, *P*<0.05, Bonferroni corrected for the number of channels) are colour-coded. Approximate areal borderlines are superimposed on the maps. (**c**) Time-frequency analysis of representative cue-evoked response in TE. (top) ERSP evoked by cue stimulus presentation. (middle) Theta component of the cue-evoked potential corresponding to the top panel. A band-pass filter of 4–8 Hz was applied to the raw signal. (bottom) Time course of power of theta activation corresponding to the middle panel. Red line indicates±2s.d. of theta power during the baseline period. ERSP, event-related spectral perturbation.

In each trial of the PA task, one of the learned visual items was presented as a cue. After a delay, monkeys were required to respond upon the presentation of the correct paired associate of the cue (Fig. 1c). ECoG recordings revealed visual cue-evoked potentials in all 128 channels (Fig. 2a and Supplementary Fig. 3). Interestingly, the waveforms of cue-evoked potentials appeared to invert between TE and other areas. Different cortical layer structures between the visual association area (TE) and the MTL may cause this polarity inversion^13^,^20^,^21^.

As a prerequisite for the analysis of spatial patterns of oscillatory ECoG responses, we examined the response selectivity of the cue-evoked power changes in theta (4–8 Hz), alpha (8–13 Hz), beta (13–30 Hz), gamma (30–70 Hz) and high-gamma (70 Hz<) frequency bands using one-way analysis of variance (ANOVA). The majority of channels distributed from TE to MTL showed significant selectivity in theta-frequency band power ∼200 ms after the cue onset in the two monkeys (Fig. 2b and Supplementary Fig. 4; one-way ANOVA; *F*_(9,1098)_=3.4 (monkey K), *F*_(9,1108)_=3.4 (monkey M), *P*<0.05, Bonferroni correction for the number of channels). Although alpha band power also exhibited significant selectivity, the spatial extent was small compared with that of the theta-band power, and few channels showed selectivity in beta-band power (Supplementary Fig. 5). We did not find significant response selectivity in either the gamma or high-gamma frequency bands during the cue period. To further analyse cue-evoked power changes in the theta-band, we conducted time-frequency spectral analysis. A representative event-related spectral perturbation plot in TE indicated a transient increase of the theta-frequency power until about 350 ms after the cue onset (Fig. 2c), the time period during which significant response selectivity of theta activity also emerged (Supplementary Fig. 4).

### Cortical theta activity patterns encode associative memory

**Figure 3:**
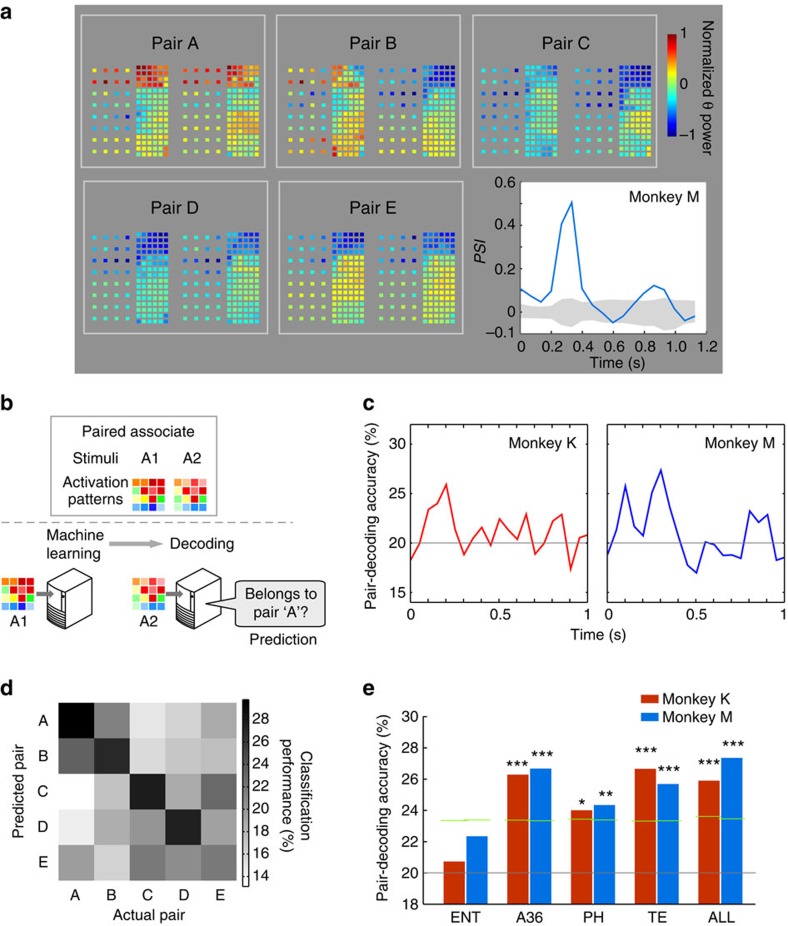
Associative-memory signature as theta pattern similarity. (**a**) Snapshots of spatial patterns of theta activity at the time of the highest *PSI* value. The normalized mean power of theta activity during a 500 ms time window centred at 332 ms after the cue onset is colour-plotted for each channel. Inset: Time course of *PSI*. Grey shading indicates the 99.99% CI corrected for the number of time points. (**b**) Schemas of the pair decoding. (top) An example of associated visual stimulus pair (A1 and A2) and their accompanying theta patterns (schematic illustrations). (bottom) A machine-learning decoder is trained on theta patterns evoked by stimulus A1, and then tested on theta patterns evoked by stimulus A2 to correctly predict the label of the test data (see also Methods). (**c**) Time courses of the pair-decoding accuracy. Peak accuracies were significantly above chance (20%, gray lines; *n*=2 monkeys, permutation test, *P*<0.001, corrected for the number of time points). (**d**) Confusion matrix of the pair decoding. The elements along the left-to-right diagonal axis correspond to correct predictions for stimuli that were actually paired together. Trials from two monkeys were merged. (**e**) The pair-decoding accuracy in each brain areas within the recording site. Brain areas other than ENT show significantly higher accuracy than the chance-level (*n*=2 monkeys, permutation test, ***P<0.001, **P<0.01, *P<0.05, corrected for multiple comparison). The short green lines indicate 5% significance levels for the permutation tests. The grey line indicates the chance-level (20%). ALL: all channels, CI, confidence interval.

We tested our hypothesis based on these results by focusing on whether similar spatial patterns of theta activity were elicited by both members of the learned paired associate. Therefore, we converted each theta pattern into a 128-dimensional vector, then calculated the ‘pairwise pattern similarity index' (*PSI*) based on the Euclidean distance across the vectors (see Methods). Significantly high *PSI* values during the cue period were revealed in the two monkeys (Supplementary Fig. 6; permutation test, 10,000 permutations, *P*<0.001, corrected, *n*=2 monkeys). Snapshots of the mean theta power maps at the peak *PSI* value demonstrate marked pairwise theta pattern similarity (Fig. 3a and Supplementary Fig. 7).

Further, we conducted a multivariate machine-learning approach that we call ‘pair decoding' to assess trial-by-trial robustness and specificity of theta pattern similarity (Fig. 3b)^22^. Initially, a machine-learning decoder was trained on theta activity patterns evoked by one member of a particular paired associate that was presented as a cue. This decoder was then tested as to whether it was able to correctly predict the identity of theta patterns evoked by the other member of the paired associate. The pair-decoding accuracy was significantly higher than chance level (20%) during ∼100–300 ms after the cue onset (Fig. 3c; permutation test, 25,000 permutations, *P*<0.01, corrected, *n*=2 monkeys), indicating a marked trial-by-trial similarity of the theta activity patterns elicited by associated visual stimuli. To demonstrate the specificity of theta pattern similarity, we constructed a confusion matrix of the pair decoding (Fig. 3d). The upper-left to lower-right diagonal elements were higher compared with the other elements, indicating the specificity of within-paired-associate similarity. To investigate functional area differences of the theta pattern similarity, we computed the pair-decoding accuracy separately in TE, A36, the ENT and the PH. Both TE and A36 exhibited significantly higher decoding accuracy compared with chance (permutation test, 5,000 permutations, *P*<0.001, corrected, *n*=2 monkeys; Fig. 3e and Supplementary Fig. 8). Moreover, inter-areal comparisons of decoding accuracy showed that there was no significant difference in decoding accuracy between TE and A36 (permutation test, Supplementary Fig. 9). Therefore, TE and A36 equally contributed to the representation of PA memory. The PH also showed significant decoding accuracy (permutation test, 25,000 permutations, *P*<0.01 in monkey M, *P*<0.05 in monkey K; Fig. 3e and Supplementary Fig. 8), but it was less significant than TE or A36. These results demonstrate that the associative-memory representation can be decodable from the LFP patterns on the cortical surface *in vivo*.

### Emergence of theta pattern similarity after novel learning

**Figure 4:**
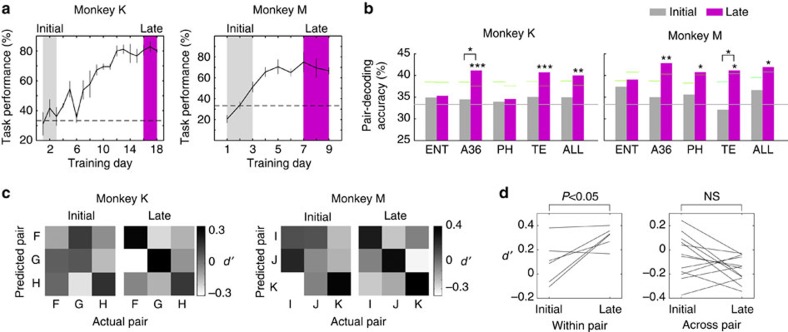
Emergence of theta pattern similarity after learning. (**a**) The learning curves of novel PA learning in monkeys K and M. Initial- and late-learning periods are shown by grey and purple bands, respectively. The horizontal dotted lines indicate a chance level of 33.3%. Error bars: s.d. (**b**) Comparisons of the pair-decoding accuracy between initial- and late-learning periods. The decoding accuracy in the late-learning period is significantly above chance in A36, TE and ALL (all channels) in monkeys K and M; in PH in monkey M (*n*=2 monkeys, permutation test, ***P<0.001, **P<0.01, *P<0.05, corrected for multiple comparison). Horizontal grey lines indicate the chance level (33.3%). Short green lines indicate 5% significance levels in the permutation tests. Also, decoding accuracy is significantly higher in the late- than in the initial-learning period in A36 (monkey K) or in TE (monkey M) (*n*=2 monkeys, permutation test, *: *P*<0.05). (**c**) Development of the theta pattern similarity. *d'* for each pair-decoding are arranged in confusion matrices (see Methods). The elements along the left-to-right diagonal axis correspond to correct predictions. The confusion matrices changed into diagonal patterns in the late period of learning in the two monkeys. (**d**) Comparisons of average *d'* between the initial- and late-learning periods. Lines indicate each of six pairs in monkeys K and M. Diagonal (within pair) and non-diagonal (across pair) elements of the confusion matrices in (c) were separately averaged, and compared between the initial- and late-learning periods. The averaged *d'* of within-pair decoding is significantly greater in the late- than in the initial-learning period (*n*=2 monkeys, two-tailed paired *t*-test, *P*<0.05).

Finally, we analysed whether the theta pattern similarity evolved through novel associative learning. We trained the two monkeys on the PA task using three novel pairs of visual stimuli (Fig. 4a). We found that A36, TE (in the two monkeys) and the PH (in monkey M) indicated significantly higher decoding accuracy than chance-level only during the late phase of learning (permutation test; Fig. 4b). Permutation tests also revealed that decoding accuracy during the late phase was significantly higher than during the initial phase in A36 (monkey K), or in TE (monkey M). The confusion matrices converted to diagonal patterns, indicating strong similarities within associated pairs after learning (Fig. 4c,d). These results suggest that the theta pattern similarity in the temporal lobe can be acquired through associative learning, probably via learning-related reorganization of neural ensembles.

## Discussion

Our high-density ECoG recordings revealed the neuronal ensembles synchronizing within the theta-frequency band as mesoscopic modules distributed across the visual association area TE to A36, the PH and the ENT of the MTL. Through learning-related reorganization, these ensembles in TE, A36 and the PH, but not in the ENT, expressed similar activation patterns specific to associated visual items, indicating a possible entire picture of associative-memory representation, beyond the analysis at the level of single neurons.

The large-scale LFP recordings provided wide coverage of the transition from the visual association area TE to the MTL memory system in the primate brain. Additional analysis confirmed that differing electrode spacing of the electrode grid did not significantly affect the decoding accuracy in the present study (Supplementary Figs 1 and 10). However, its current spatial resolution is insufficient to resolve brain activation patterns at the level of a single cortical column, known as an important functional unit in the primate cerebral cortex. Despite this limitation, our results demonstrate that area-wide distribution of LFP patterns generated by hypercolumnar-scale mesoscopic neuronal ensembles can effectively encode declarative memory in the macaque brain. This view is consistent with recent studies showing that various cognitive and mental states can be decoded from brain activation patterns in animals and humans^19^,^23^,^24^,^25^. To further clarify the functional roles of the theta activation patterns, it would be interesting to compare theta patterns in correct and incorrect trials, but we were not able to conduct such an analysis in the present study, because there was not a high enough number of incorrect trials to be subjected to the analysis.

A series of single-unit recording studies of monkeys performing PA tasks identified cortical singular spots in the temporal lobe containing ‘pair-coding neurons,' which exhibit spiking activity selective to learned visual associations^6^,^9^,^10^,^11^,^12^. These spots have been shown to predominantly localize within a restricted region in A36 (refs 10, 12). Our study revealed that the theta activity patterns are broadly distributed across TE and A36, and these areas equally exhibit significantly high pair-decoding accuracy, indicating that the singular spots containing pair-coding neurons are embedded within widespread theta patterns representing associative memory. Electrophysiological studies have suggested that neuronal spike activity may be coupled to higher frequency gamma-band LFP activity, whereas lower frequency activity is thought to be involved in the broader neuronal network^26^,^27^. Therefore, it is unlikely that the theta patterns representing associative memory are directly coupled to the spike activity of the pair-coding neurons. Previous studies have identified both bottom-up and top-down information flow into pair-coding neurons^6^,^9^,^10^,^12^,^28^. The theta patterns revealed in the present study may be manifestations of two-dimensional profiles of information flow specific to each paired-associate. An important question for future studies is to determine relationships between the spatially distributed theta patterns and the spike activity of the pair-coding neurons.

Our results also demonstrated the contribution of the theta patterns in the PH in the representation of associative memory, though it was less significant than in TE and A36. Such area differences in significance may be based on differences in recording areal coverage: relatively small portion of the PH was recorded compared with TE and A36 in the present study (Fig. 1b). Although the PH has been shown to process spatial information, several studies have indicated that the PH is also implicated in the processing of object information, especially in terms of contextual association^29^,^30^,^31^,^32^. This notion is also supported by the present results.

Hargreaves *et al*.^14^ conducted tetrode recordings in both the ENT and HC of monkeys during stimulus-response associative learning. They reported that the beta-band LFP power gradually increased in the ENT as associative learning progressed, indicating possible contributions of the ENT to associative learning. In the present study, preliminary observations indicated tonic activity of theta to beta frequency bands during the delay period of the task trial (Fig. 1c) in the ENT. In addition, the power of the delay activity was greater in the late phase than in the initial phase of the associative learning (data not shown). Although these preliminary observations are largely consistent with the findings of Hargreaves *et al*., our pair-decoding analysis indicated that the spatial patterns of activation during the cue period were not significantly similar for the paired associates learned through stimulus–stimulus associations in the ENT (Fig. 3e). Therefore, our results suggest that the ENT may play different roles in processing associative memory signals than the A36, PH and TE.

It has been shown that the HC is involved in the formation of both spatial and non-spatial explicit memory in rodents, humans and non-human primates^1^,^33^,^34^, where tonic theta-band oscillation often plays a crucial role in coordinating information processing^35^,^36^,^37^,^38^. Although the transient nature of theta responses shown in the present study is clearly distinctive from the tonic theta oscillation observed in the HC, it is possible that activation in the theta-frequency may be one of the common ways of representing explicit memory in the MTL. The current study may thus provide a possible unifying view of the MTL memory system via theta rhythm.

## Methods

### Subjects

Two female macaques (*Macaca fuscata*; monkey K, 11 years of age, 4.1 kg; monkey M, 6 years of age, 5.1 kg) were used. All animal procedures complied with the National Institute of Health Guide for the Care and Use of Laboratory Animals, and with the Guide of the National BioResource Project ‘Japanese Monkeys' from MEXT, Japan. The Niigata University Institutional Animal Care and Use Committee approved the experimental protocols.

### Task procedures

The subjects were trained on a sequential version of the visual PA task. Five pairs (A1:A2 to E1:E2) of visual stimulus–stimulus associations were learned by the two monkeys. In each task trial, when the subjects held a lever, a fixation point (0.1 × 0.1°, square) appeared at the centre of a monitor for 1–1.5 s. Then, one of the visual stimuli was presented as a cue for 1 s. After a 1.5 s delay period, up to three choice stimuli were sequentially presented (0.3 s each) at 0.6 s intervals. The choice stimulus was either the paired associate of the cue or a distractor randomly selected from a different pair. When the subjects released the lever upon the presentation of the correct paired associate, a drop of juice was delivered as a reward. In the PA task, both members of a given paired-associate could be used as a cue. For example, the monkeys had to recall stimulus A2 when stimulus A1 was presented as a cue, and also had to recall stimulus A1 when stimulus A2 was presented as a cue. Visual stimuli (4 × 4°) were presented on a 17-inch LCD monitor (EIZO, Ishikawa, Japan). The monkeys' heads were restrained with a titanium head holder (Gray Matter Research, MT, USA), which was attached to the skull by standard operation procedures with appropriate postoperative care. Gaze positions were monitored using an infrared camera system at a sampling rate of 60 Hz (http://staff.aist.go.jp/k.matsuda/eye/), and central gaze fixation (±1°) onto the fixation point overlying the visual stimuli was required throughout a trial. Task controls and behavioural data recordings were performed using custom-made software (NSCS, Niigata, Japan) running on LabVIEW Real-Time (National Instruments, TX, USA).

### Electrode grid for ECoG recordings

We designed a 128-channel electrode grid covering an area of 16 × 20 mm for recording from the monkey's temporal lobe (Supplementary Fig. 1a,b). The electrode grid was fabricated on a 20-μm-thick flexible Parylene-C film using micro-electro-mechanical systems technology^15^,^16^. A 0.5 × 0.5 mm square of the gold surface was exposed at each electrode tip position (Supplementary Fig. 1a,b).

### Electrode grid implantation

The electrode grid was subdurally implanted onto the left inferior to medial temporal lobes of the two monkeys^15^. All surgical procedures were conducted under aseptic conditions. Monkeys were initially premedicated with ketamine (50 mg kg^−1^ bodyweight (BW)) and medetomidine (0.03 mg kg^−1^ BW). Then, each monkey was intubated with an endotracheal tube and connected to an artificial respirator (ADS 1000, Engler Engineering Corp., FL, USA). Anaesthesia was maintained with inhalation of Isoflurane (1.5–2.5% in oxygen). Heart rate, oxygen saturation and end-tidal CO2 were continuously monitored (SurgiVet, Smiths Medical, London, UK) to adjust the anaesthesia level. Careful postoperative treatment including daily delivery of antibiotics (cefazolin, 20 mg kg^−1^ BW, intramuscularly (i.m.)) and analgesic (ketoprofen, 20 mg, i.m.) for a week was provided. A custom-made, Mayfield-type head clamp was used to obtain access to the temporal bone. The target location and the size of craniotomy were determined using preoperative magnetic resonance imaging. After the craniotomy, the dura mater was cut with scissors into four quadrants, and the entire anterior middle temporal sulcus (amts) and anterior region of the superior temporal sulcus (sts) were identified under an operating microscope (Carl Zeiss, Oberkochen, Germany). The electrode grid was carefully attached onto the cortical surface over the amts, which was used as a landmark to closely match the configuration of the electrode grid between the two monkeys (Fig. 1b and Supplementary Fig. 1c,d). The electrode was covered with a piece of Gore-Tex film (W. L. Gore & Associates, DE, USA) to prevent adhesions before closing the dura. Dural defects were patched using the fascia of the temporal muscle with watertight suturing to prevent cerebrospinal fluid leakage. The electrode lead, microconnectors (Omnetics, MN, USA), and a custom-made plastic connector chamber (Vivo, Hokkaido, Japan) were fixed onto the bone with resin. Layer-by-layer suturing was performed to complete the operation.

### ECoG recordings

ECoG signals were amplified using a 128-channel differential amplifier (Plexon, TX, USA) with band-pass filtering (0.7–300 Hz). All subdural electrodes were referenced to a titanium head-restraint post that contacted the dura via multiple titanium bolts. Electrode impedance was typically 20–50 kΩ. Signals were stored at a sampling rate of 1 kHz per channel onto a hard disc controlled by LabVIEW Real-Time (National Instruments, TX, USA) using custom software.

### Histology

Histological analysis was performed by conventional methods^39^. After the recording experiments were completed, monkeys were deeply anaesthetized with a sodium pentobarbital overdose and transcardially perfused with 4% paraformaldehyde in 0.1 M PBS (pH 7.4). Brains with the electrode grid attached were removed from the skull. The left temporal lobe of each monkey was cut into a block containing the recording site, and the positions of the electrode array were marked onto the cortex using fluorescent dye^40^. The blocks were then cryoprotected in 20, 25 and 30% sucrose in 0.1 M PB at 4 °C until they sank. These blocks were sectioned into 40 μm coronal slices using a cryostat (Leica Biosystems, Nussloch, Germany), then collected into five series, and mounted onto slides. One series of sections was stained for Nissl with cresyl violet and coverslipped with Permount (Fisher Scientific, NJ, USA). The stained sections were photographed with a microscope (BZ-9000, Keyence, Osaka, Japan). Borders between each brain region were determined according to the cytoarchitectonic criteria described in previous studies^20^,^21^. Major criteria we used were as follows. For the border between A36 and TE: layers V and VI in TE are more clearly separated than those in A36; in the rostral part, cell aggregates are observed in layer II of A36, but not in TE. For the border between the PH and TE: neurons in TE are oriented more radially than those in the PH; layers IV, V and VI appear relatively homogeneous in the PH compared with those in TE; the signals of immunohistochemistry for PV are greater in TE than in the PH. The ENT is identified as an agranular cortex.

### Immunohistochemistry

Immunohistochemistry for PV was performed according to a protocol published by other researchers with a slight modification^21^. Free-floating sections were first incubated with 3% H_2_O_2_ in phosphate-buffered saline (PBS, pH 7.4–7.6) for 1 h to quench endogenous peroxidase, then washed in PBS for 10 min. After preincubation with 0.5% Triton X-100 and 5% normal goat serum in PBS for 60 min at RT, the sections were incubated with the primary antibody (anti-PV monoclonal, PARV-19, Sigma-Aldrich, 1:2,000) in 0.5% Triton X-100 and 5% normal goat serum in PBS for 2 days at 4 °C. After washing in PBS for 30 min three times, the sections were incubated with the secondary antibody (anti-mouse IgG conjugated with HRP, Jackson, 1:400) in 0.5% Triton X-100 and 5% normal goat serum in PBS for 90 min at RT. After washing in PBS for 30 min three times, the sections were reacted with 0.05% diaminobenzidine and 0.003% H_2_O_2_ in 0.05M Tris buffer (pH7.2–7.4). The stained sections were mounted on slides, and were photographed using a microscope (BZ-9000, Keyence, Osaka, Japan).

### Data analysis

All analyses were performed using MATLAB (MathWorks, MA, USA) with in-house code, the Statistics and Signal Processing Toolboxes (MathWorks), and an open-source toolbox, EEGLAB (http://sccn.ucsd.edu/eeglab/)^41^.

### Data preprocessing

The ECoG responses on correct task performance during the 3-day recording sessions were analysed. In total, 1,108 and 1,118 correct trials (for monkeys K and M, respectively) were used. For the ECoG recording experiment across the initial- and late-learning periods, 609 (monkey K) and 286 (monkey M) trials including both correct and incorrect trials during initial periods, and 597 (monkey K) and 296 (monkey M) correct trials during late period were analysed. Sample size was determined based on existing researches in the field. Noise components were eliminated using the independent component analysis implemented in EEGLAB. The resultant ECoG responses were aligned at the onset of cue presentation, defined as time=0, and averaged for each cue stimulus with offset correction against the mean baseline-amplitude (–50 to 0 ms).

### Time-frequency spectral analysis

The wavelet algorithm implemented in EEGLAB was used for time-frequency spectral analysis. The power spectra of ECoG responses were calculated for each 550 ms time-bin, sliding from time=−500 to 2,500 ms with a 24.7 ms step. The averaged power before the cue onset (–500 to 0 ms) was used for baseline offset correction.

### Stimulus selectivity

To assess the selectivity of spectral responses to the stimuli, the mean power of the theta (4–8 Hz), alpha (8–13 Hz), beta (13–30 Hz), gamma (30–70 Hz) and high-gamma (70 Hz<) activity within a 270-ms time window was subjected to one-way ANOVA. To determine the time course of stimulus selectivity, the time-window was shifted from −500 to 2,500 ms with 27.5 ms-steps. *F*-values greater than the significance level were reported (*P*<0.05, with Bonferroni correction for both the number of channels and time windows).

### Pairwise pattern similarity index (*PSI*)

We introduced a ‘*PSI*' based on Euclidean distance to evaluate the theta pattern similarity. Channel-wise theta activity (4–8 Hz) power was converted into the population vectors **V**_*i*_ and 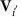, where **V**_*i*_ represents the population vector for stimulus *i*, and 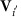 represents the population vector for the paired-associate of stimulus *i.* Euclidian distances between the vectors of each paired-associate were calculated and averaged as follows:


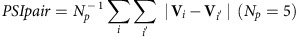


Then, **V**_*k*_ represents the population vectors for the stimuli that are not the paired-associate of stimulus *i.* For a baseline, Euclidian distances between vectors of combinations other than learned paired associates were calculated and averaged as


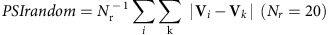


*PSI* was defined as follows:


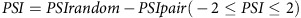


Higher *PSI* values correspond to higher degrees of similarity between the activity patterns evoked by each paired associate compared with baseline similarities across random pairwise combinations of activity patterns. *PSI* was calculated within a 500 ms time window shifted at a 66 ms step. We assessed the statistical significance of *PSI* using a permutation test in which the labels of each theta pattern were shuffled 10,000 times (*P*<0.001, with Bonferroni correction for the number of time windows).

### Pair decoding

To evaluate theta pattern similarity on a trial-by-trial basis, we conducted a multivariate machine-learning approach termed ‘pair decoding.' For pair decoding, patterns of theta activity of all 128 channels within a 270-ms time window and their labels were entered into a linear support vector machine implemented in LIBSVM (http://www.csie.ntu.edu.tw/~cjlin/libsvm)^42^. This decoder was then tested on theta patterns evoked by the paired-associate presented as a cue in predicting its identity. For example, a decoder was trained on ECoG patterns evoked by stimuli A1, B1, C1, D1 or E1 (training data). Each training datum was labelled as its pair name, for example, ECoG patterns evoked by A1 were labelled as ‘A' (not ‘A1'). Then, as test data, ECoG patterns evoked by A2, B2, C2, D2 or E2 were entered into the decoder in a trial-by-trial manner. The decoder predicted the pair-name label of each test datum. For example, if the decoder predicted label ‘A' when an ECoG pattern evoked by A2 was entered, the prediction was correct. Decoding accuracy *Af* here was calculated as


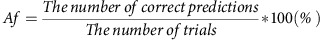


A decoder was next trained on ECoG patterns evoked by A2, B2, C2, D2 or E2 (training data), and then predicted labels of ECoG patterns evoked by A1, B1, C1, D1 or E1. Decoding accuracy *Ab* here was calculated as:


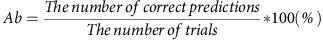


Final decoding accuracy was reported as


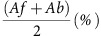


Note that training and test data sets never overlapped in our pair-decoding. The decoding accuracy was first averaged within each pair and then across pairs. To obtain time courses of decoding accuracy, the time window was shifted from time=0 to 1,000 ms in 50-ms steps. We applied this procedure to all paired associates. We assessed the statistical significance of decoding accuracy using a permutation test that shuffled the labels of each theta pattern 25,000 times (*P*<0.01, with Bonferroni correction for the number of time windows). These procedures were also conducted separately in channels in area TE, A36, the ENT and the PH. The criteria for channel classifications into each brain region were solely based on cytoarchitectures as revealed by the histology (Fig. 1b and Supplementary Fig. 2). We also performed pairwise comparisons of decoding accuracy across the brain regions by permutation tests; we obtained a surrogate distribution of differences of decoding accuracy between two given brain regions of comparison by 5,000 times permutation (Supplementary Fig. 9). We determined statistical significance by comparing the surrogate distribution and the actual difference. We corrected *P* values with the number of pairwise comparisons (pairwise comparisons across TE, A36, PH and ENT: _4_C_2_=6).

### Confusion matrix

A confusion matrix at the time point of peak pair-decoding accuracy during the cue period was constructed and merged across the two monkeys (Fig. 3d). The proportions of trials classified as the pairs on the vertical axis (predicted pair: row) were plotted against the actual trials derived from the horizontal axis (actual pair: column). Each sum of columns was normalized to 100%.

In the novel associative learning experiments, confusion matrices were constructed using *d'* for each pair-decoding accuracy (Fig. 4c). We calculated the true positive rate (*TPR*) and false positive rate (*FPR*) for each decoding as follows:


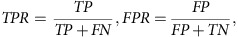


where *TP*, *FN*, *FP* and *TN* indicate the number of true positive (hit), false negative (miss), false positive (false alarm) and true negative (correct rejection) in each decoding, respectively. *TPR* and *FPR* were converted into *Z*-scores, *Z*(TPR) and *Z*(FPR), using the normal inverse cumulative function, and *d′* was defined as:


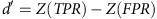


### Novel associative learning experiment

The two monkeys learned three additional pairs of visual stimulus–stimulus associations (pairs F–H and I–K for monkeys K and M, respectively). Each training day (TD) consisted of 15–20 sessions, and each session was composed of 36 trials. On each TD, performance was measured by averaging the top five performances among sessions, and plotted to obtain the learning curves. ECoG recordings were conducted during initial (TD 1–4 and 1–3 for monkeys K and M, respectively) and late (TD 17–19 and 7–9, monkeys K and M, respectively) learning periods.

### Data availability

The data that support the findings of this study are available from the corresponding authors upon request.

## Additional information

**How to cite this article:** Nakahara, K. *et al*. Associative-memory representations emerge as shared spatial patterns of theta activity spanning the primate temporal cortex. *Nat. Commun.* 7:11827 doi: 10.1038/ncomms11827 (2016).

## Supplementary Material

Supplementary Information Supplementary Figures 1 - 10
