# Supplementary material for: Associative-memory representations emerge as shared spatial patterns of theta activity spanning the primate temporal cortex
Source: Nat Commun. 2016 Jun 10;7:11827. doi: 10.1038/ncomms11827 (PMC4906394; doi:10.1038/ncomms11827)
Supplement: Supplementary Information — Supplementary Figures 1 - 10 [file ncomms11827-s1.pdf]

Supplementary Figures

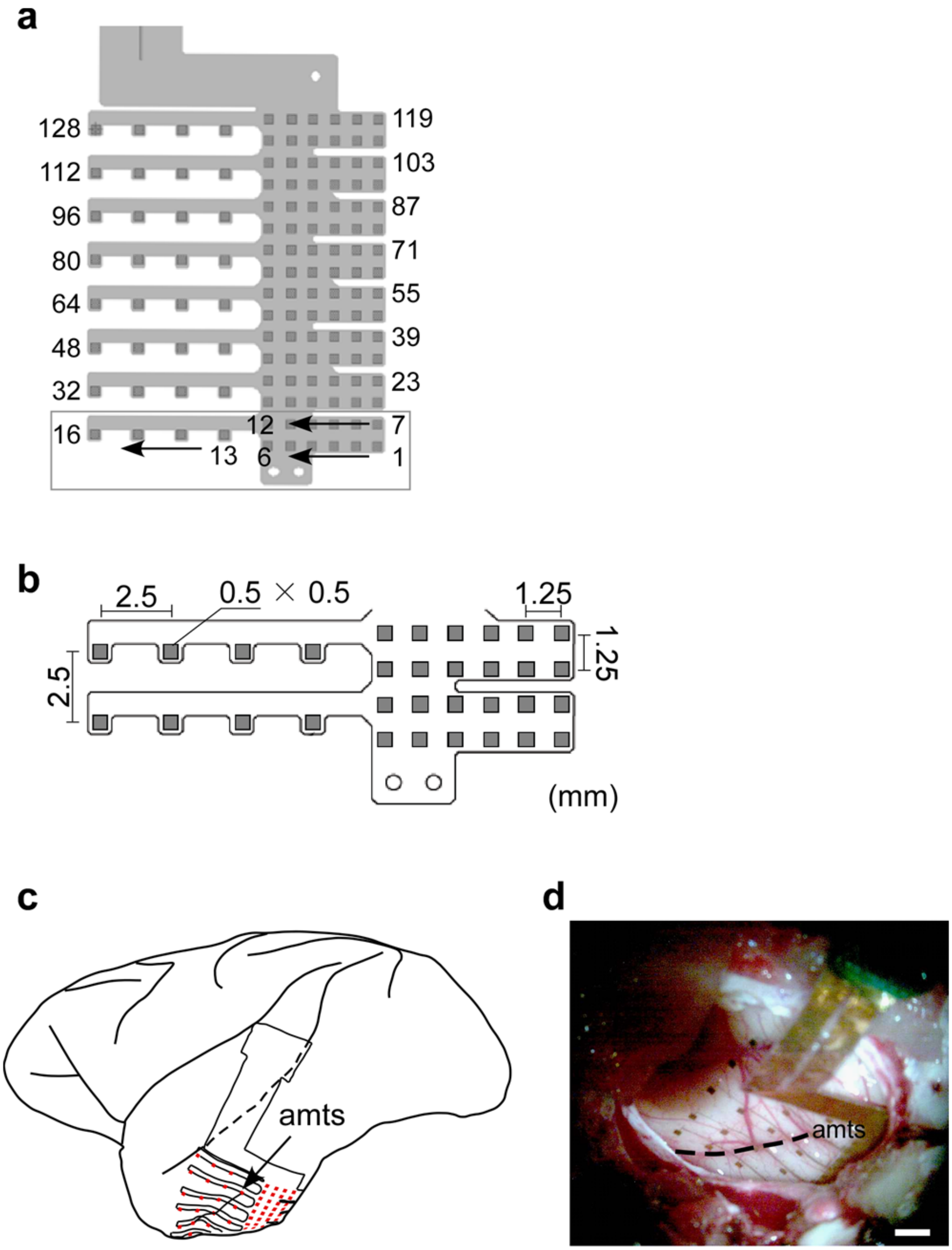

Supplementary Figure 1. Ultra-thin 128-channel electrode grid and electrode grid implantation.

(a) Channel number assignment (#1 – #128).

(b) Boxed section in (a) is magnified. Gray squares indicate each electrode tip (0.5 × 0.5 mm). Inter-electrode interval is 1.25 or 2.5 mm.

(c) Left lateral view of monkey brain implanted with a 128-ch electrode grid over the anterior middle temporal sulcus (amts). Red dots indicate each electrode channel.

(d) An operation-microscope photograph of electrode grid implantation. Each electrode channel appears as a small gold square attached onto the cortical surface. The dotted line indicates the entire amts. The electrode configuration relative to the amts was closely matched across the two monkey subjects. Scale bar: 2.5 mm.

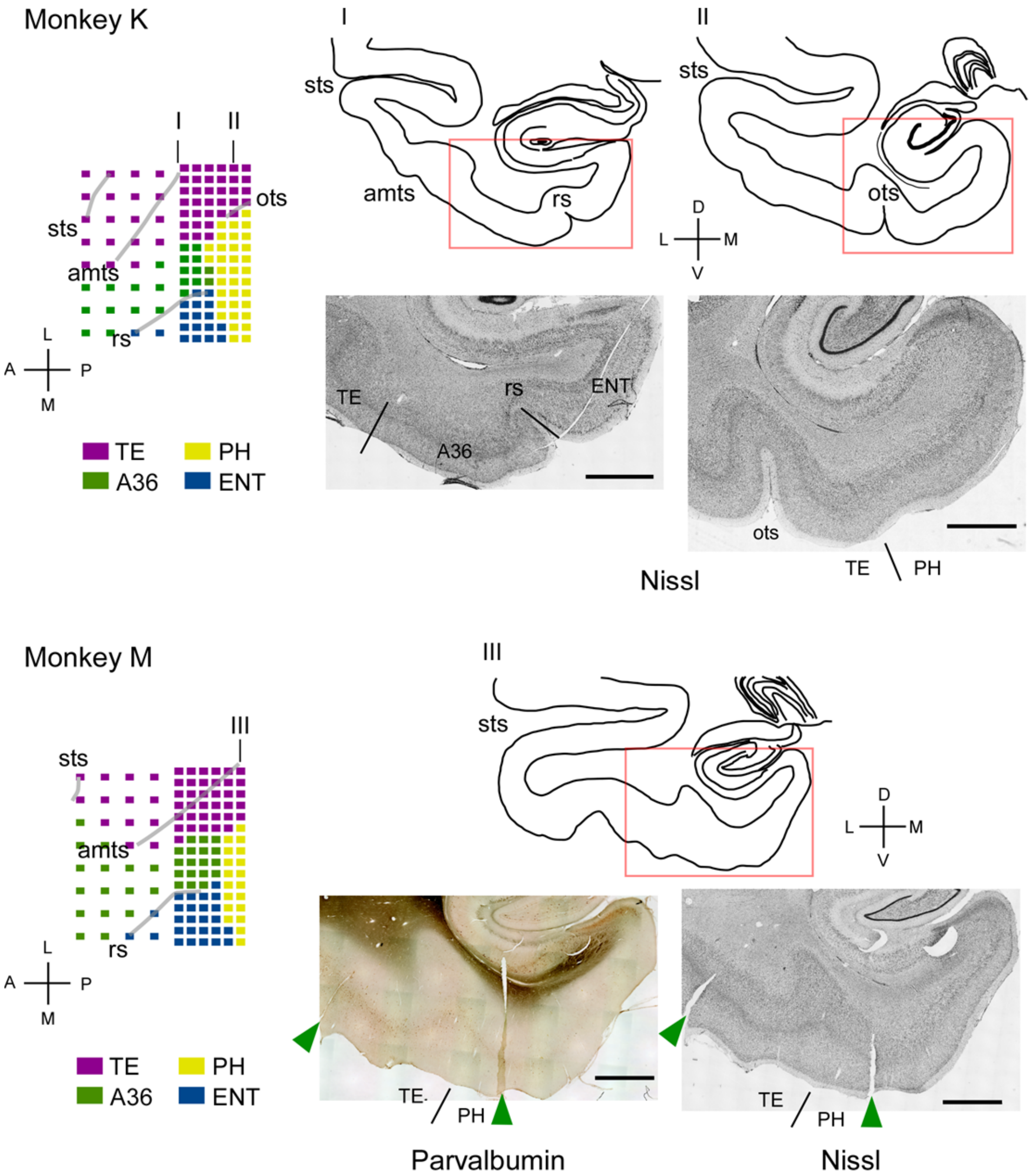

Monkey K
I
II
sts
amts
ots
rs
L
A
P
M
D
V
TE
A36
PH
ENT
Nissl
Monkey M
III
Parvalbumin

Supplementary Figure 2. Histological analysis and areal boundaries within the recording site.

(Left panels) Classifications of anatomical locations of electrode channels. Areal boundaries were determined based on the cytoarchitectural criteria[19, 20]. Lines I, II, and III indicate the levels of coronal sections shown in the right panels. amts: anterior middle temporal sulcus, ots: occipitotemporal sulcus, rs: rhinal sulcus, sts: superior temporal sulcus. TE: area TE, A36: area 36, PH: the parahippocampal cortex, ENT: the entorhinal cortex. A-P: anteroposterior, M-L: mediolateral. **(Right panels)** Nissl staining and parvalbumin (PV) immunohistochemistry (IHC). Coronal sections at the levels of I, II, and III in left panels were reconstructed with line drawings. Photographs of Nissl staining or PV IHC correspond to red boxes on the line drawings. There is a good correspondence between the change of strength of PV signals and the border of TE and PH[19, 20]. Green arrowheads on the panels of monkey M point to tracks marked on the cortex, which were used as references to reconstruct the location of electrode array on the brain. D-V: dorsoventral, M-L: mediolateral. Scale bars: 2 mm.

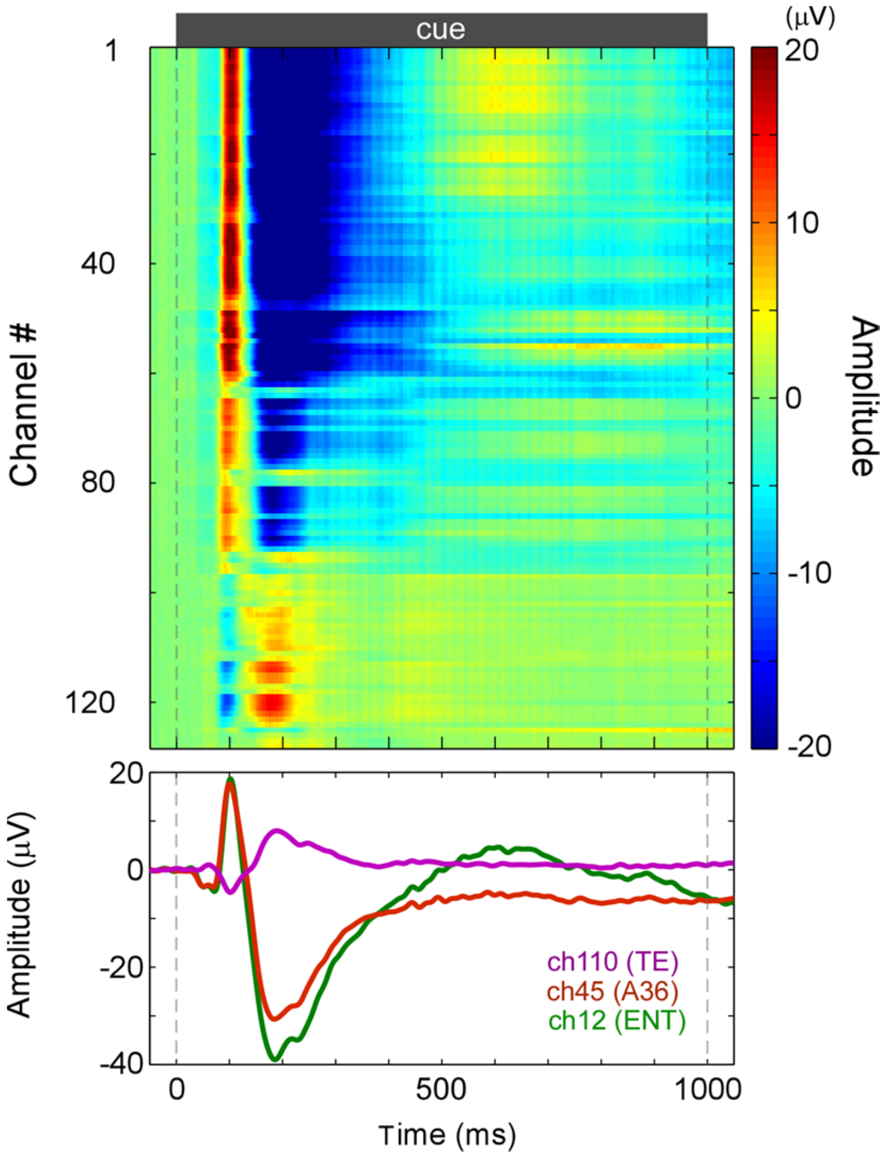

Supplementary Figure 3. Stimulus-evoked ECoG potentials in monkey K.

(Top) Amplitudes of averaged responses at each of the 128 channels during the cue period. Channel number assignments are shown in Supplementary Fig. 1. (Bottom) Representative waveforms of cue-evoked LFPs. ENT: the entorhinal cortex.

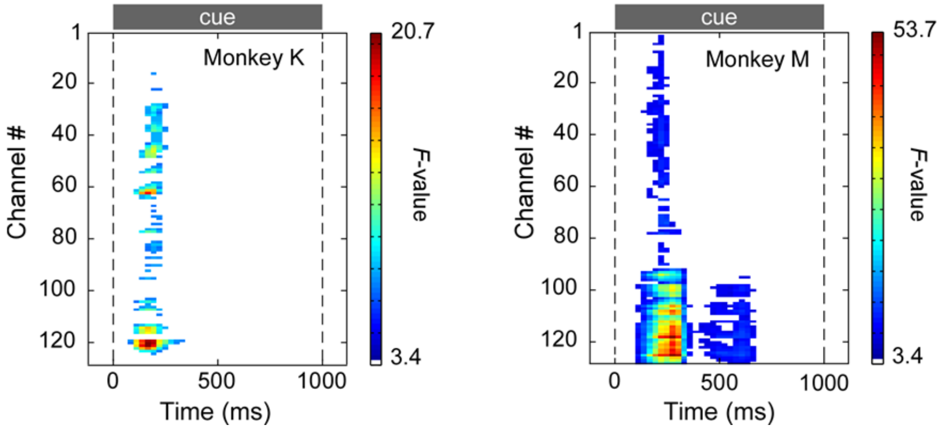

Supplementary Figure 4. Channel-wise time course of response selectivity of theta oscillation to cue stimuli. The time period from −500 ms to 2,500 ms (time = 0 was at the cue onset) was divided into 1,000 time-windows (250 ms). Theta oscillation power at each time window of each channel was subjected to ANOVA, and *F*-values above the significance level are color-coded (n = 2 monkeys, one-way ANOVA, $P < 0.001$, Bonferroni correction).

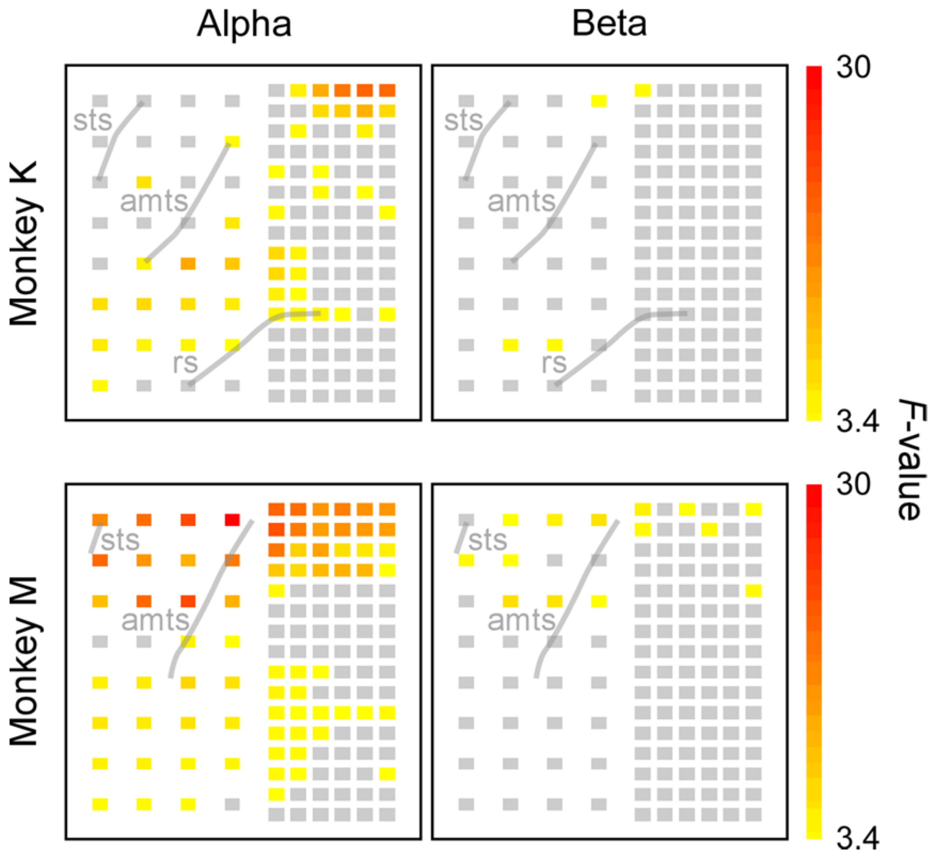

Supplementary Figure 5. Channel-wise mappings of response selectivity of alpha or beta activity to the cue stimuli.

The power of alpha or beta activity during the time period from 100 to 272 ms after the cue onset was subjected to one-way ANOVA. *F*-values above the significance level of selectivity to the cue stimuli are color coded ($n = 2$ monkeys, one-way ANOVA, $P < 0.001$, Bonferroni corrected for the number of channels). amts: anterior middle temporal sulcus, rs: rhinal sulcus, sts: superior temporal sulcus.

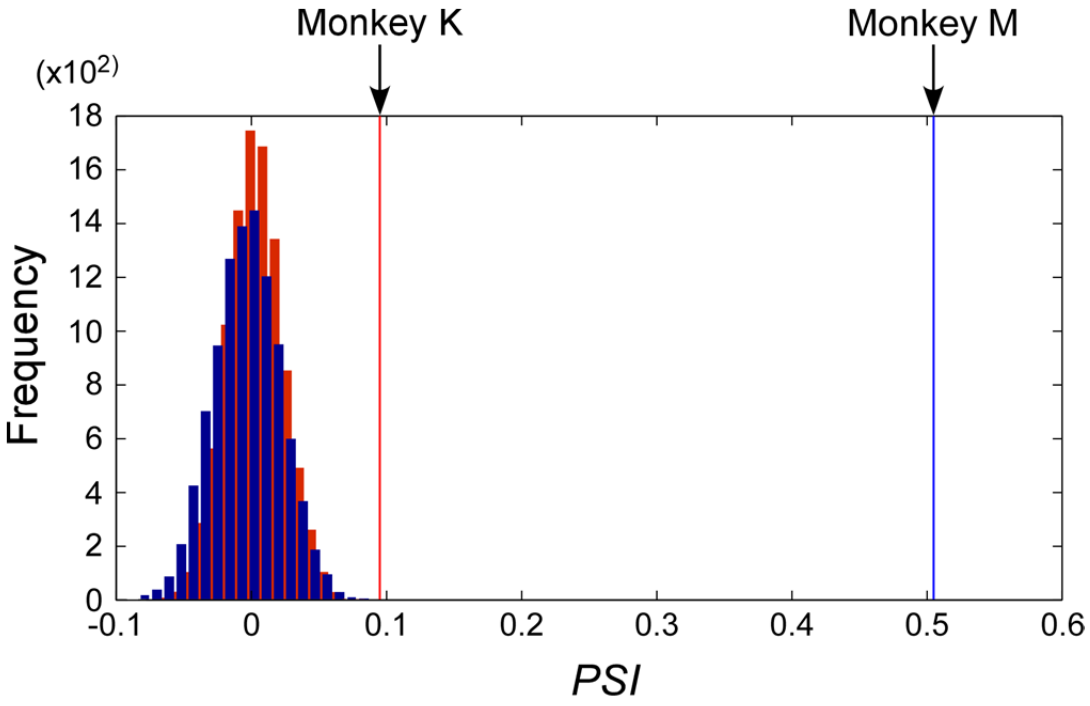

Supplementary Figure 6. Permutation tests on pairwise pattern-similarity index (*PSI*). Surrogate distributions of *PSI* for monkeys K (red) and M (blue) are shown. These distributions are calculated using 10,000 pairings with random permutation labeling. Real *PSI* values calculated with learned paired-associates are significantly separated from the surrogate distributions, which are indicated by colored vertical lines for monkeys K (blue) and M (red) (n = 2 monkeys, permutation test, $P < 1.43e^{-4}$ for monkey K, $P < 3.92e^{-128}$ for monkey M).

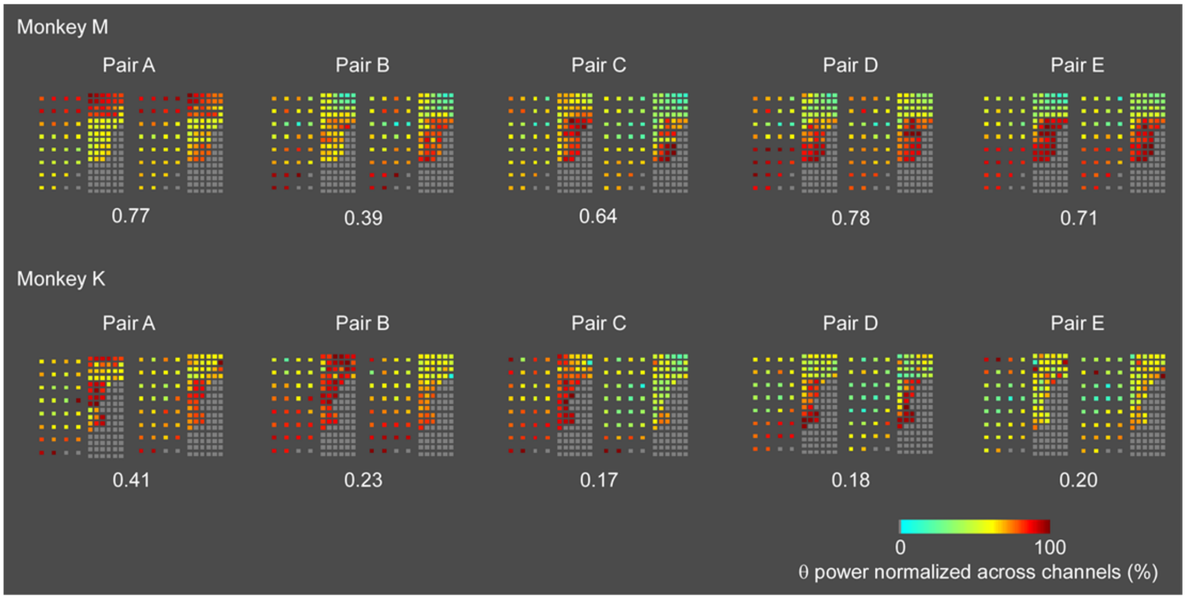

Supplementary Figure 7. Theta-power similarity maps in TE and A36 evoked by associated stimuli.

Spatial patterns of theta power at the time of the highest Pairwise Pattern-Similarity Index (*PSI*) value for each pair. Actual values of *PSI* are indicated below each pair of maps.

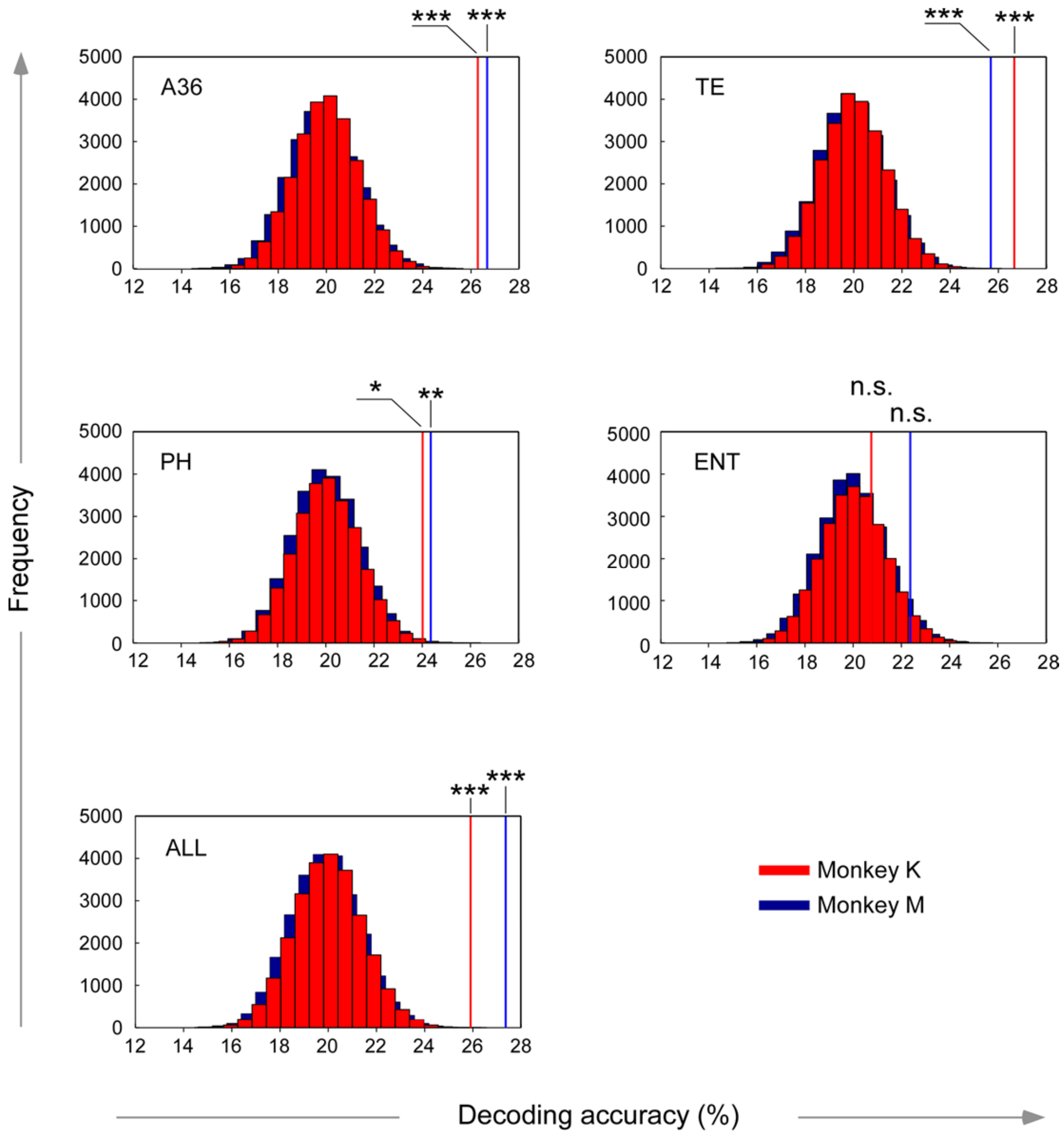
A36
TE
PH
ENT
ALL
***
**
*
n.s.
Frequency
Decoding accuracy (%)
Monkey K
Monkey M

Supplementary Figure 8. Statistical significance of the pair-decoding accuracy.

Surrogate distributions of the accuracy of pair-decoding were separately generated in each cortical area. Each distribution was calculated using 25,000 pairings with random permutation labeling. The real accuracies of pair-decoding calculated with learned paired-associates (red or blue vertical lines) are significantly separated from the surrogate distributions in A36, TE, the parahippocampal cortex (PH), and all channels (ALL), but not in the entorhinal cortex (ENT) (n = 2 monkeys, permutation test, ∗∗∗: $P < 0.001$, ∗∗: $P < 0.01$, ∗: $P < 0.05$).

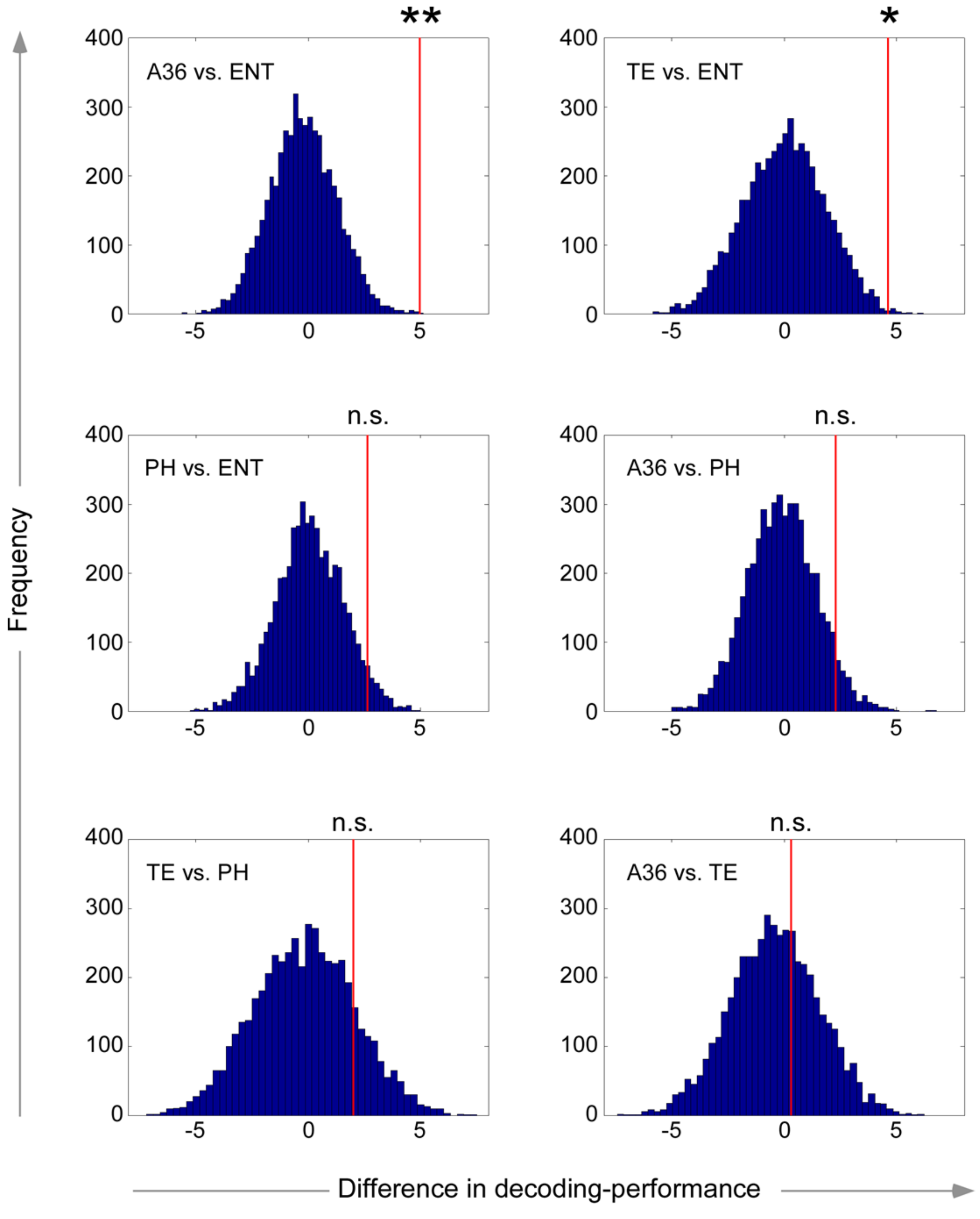
**
*
A36 vs. ENT
TE vs. ENT
n.s.
n.s.
PH vs. ENT
A36 vs. PH
n.s.
n.s.
TE vs. PH
A36 vs. TE
400
300
200
100
0
-5
0
5
Frequency
Difference in decoding-performance

Supplementary Figure 9. Inter-areal differences of the pair-decoding accuracy.

Surrogate distributions of differences in the decoding accuracy between any given two brain regions were generated by 5,000 times random permutation. Statistical significance was obtained by comparing actual differences of the decoding accuracy and the surrogate distributions. Only A36 vs. the entorhinal cortex (ENT), and TE vs. ENT exhibited statistically significant differences (n = 2 monkeys, permutation test, **: $P < 0.01$, *: $P < 0.05$). PH: the parahippocampal cortex.

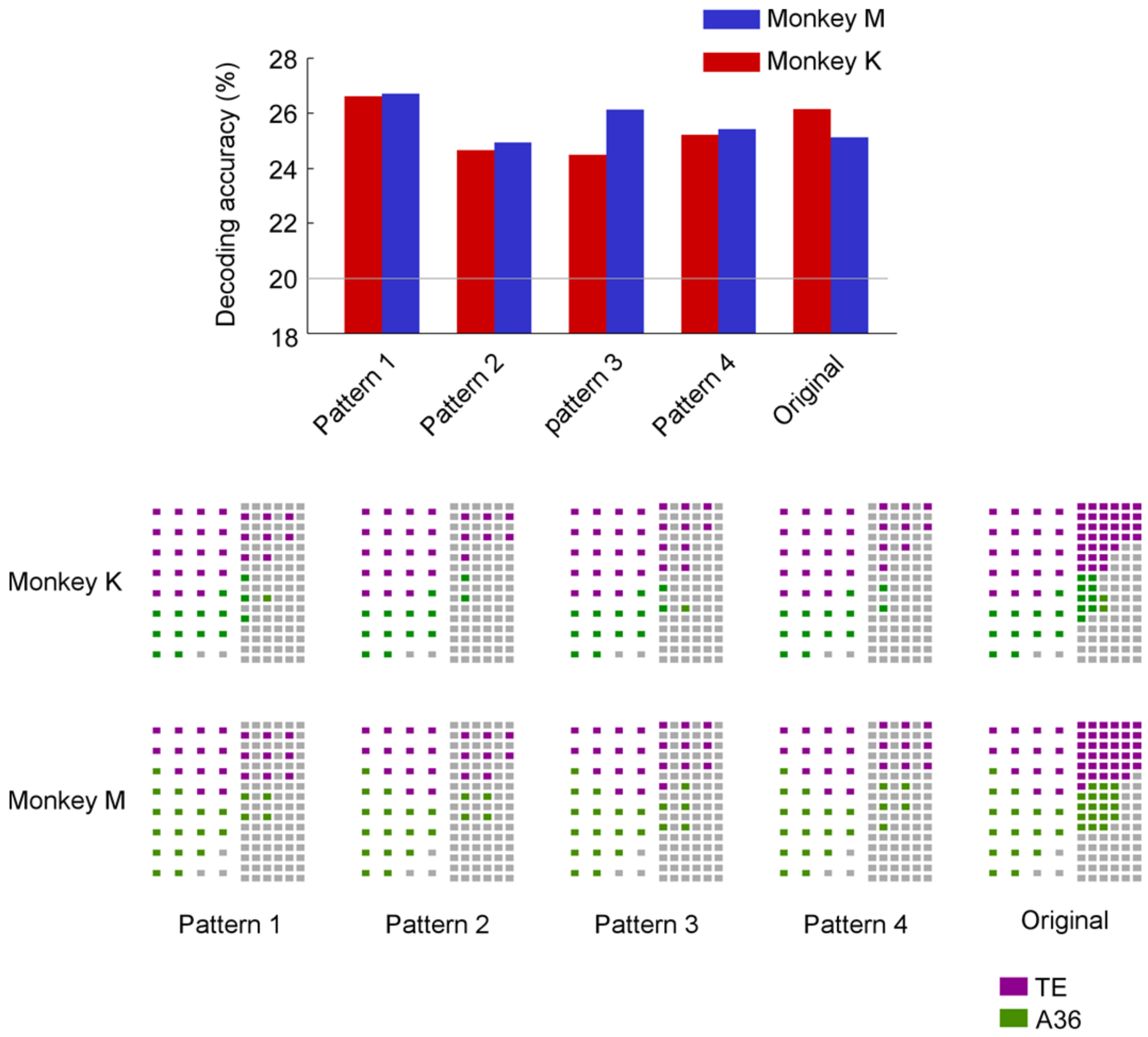

Supplementary Figure 10. Robustness of the pair-decoding accuracy against sparse sampling of the electrode channels.

The pair-decoding accuracy in TE and A36 was calculated using four different sparse sampling patterns (Pattern 1 to 4) in each monkey. Permutation tests revealed no significant differences in the decoding accuracy across patterns, indicating the robustness of pair-decoding accuracy against the sparseness of electrode channels (n = 2 monkeys, permutation test, P > 16).
